# Age and annual growth rate cause spatial variation in body size in *Phrynocephalus przewalskii* (Agamid)

**DOI:** 10.1002/ece3.7013

**Published:** 2020-11-13

**Authors:** Wei Zhao, Yangyang Zhao, Rui Guo, Yue Qi, Xiaoning Wang, Na Li

**Affiliations:** ^1^ Gansu Key Laboratory of Biomonitoring and Bioremediation for Environmental Pollution School of Life Sciences Lanzhou University Lanzhou China; ^2^ College of life sciences Hainan Normal University Haikou China

**Keywords:** age, Allen's rule, annual growth rate, Bergmann's rule, *Phrynocephalus przewalskii*, temperature

## Abstract

Whether or not biogeographic rules dealing with spatial patterns of animal body sizes are valid for ectotherms is controversial. As the ectotherms grow all their lives, we explored the role of age and annual growth rate in body size variation in *Phrynocephalus przewalskii* in northern China. Morphological data were collected from 11 populations across a broad geographic gradient. Correlations between age, sex, climatic factors, and body size were analyzed using generalized linear model (GLM) and generalized linear mixed model (GLMM). GLM analysis indicated that the general body size of both sexes and the appendage size of females increased significantly with increasing temperature; however, the coefficient of determination was very small. GLMM analysis indicated that body size only correlated with age, whereas appendage size was affected by age, temperature, rainfall, and sunshine. Annual growth rates were positively correlated with temperature. We concluded that body size variation was mainly caused by age structure and plasticity of the growth rate in *P. przewalskii* and did not follow Bergmann's rule; however, females followed Allen's rule. Future studies to investigate the effect of energy restriction are needed to further understand the relationship between growth rate and body size. We also suggest that further studies on thermal advantage and sexual selection may be helpful to understand appendage size variation in *P. przewalskii*.

## Graphical abstract

This study explores the validity of Bergmann's rule and Allen's rule in *Phrynocephalus przewalskii*, a small sand lizard prevalent in the deserts of northern China. Analysis of correlations between climatic factors and body size enabled us to conclude that body size trends were mainly caused by plasticity of growth rates, whereas appendage size trends were due to heat balance in females. We believe that our study makes a significant contribution to literature because we have shown that age should be considered, along with general body size, when testing biogeographic rules; we also recommend the use of temperature instead of latitude or altitude in such studies.

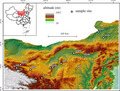

## 1 INTRODUCTION

Biogeographic rules, such as Bergmann's and Allen's rules that describe the biogeographic patterns of animal body size, have been studied and debated for over a century (Meiri, 2011; Watt et al., 2010). Bergmann proposed that larger endothermic animals with a relatively small external surface have an advantage in heat conservation over smaller relatives; hence, these animals should be found in colder areas (Bergmann, 1847; Meiri, 2011). The rule was then tested broadly, and plenty of supporting evidence was collected from endothermic animals (Ashton et al., 2000; Salewski & Watt, 2017). However, when geographic patterns are extended to interspecies or intraspecies variation of ectotherms, the situation becomes complicated and controversial (Ashton & Feldman, 2003; Jaffe et al., 2016; Jin et al., 2013; Liu et al., 2018; Olalla‐Tárraga & Rodríguez, 2007). Based on the same heat conservation mechanisms, Allen's rule, which was initially applied to endotherms, supports the general decrease in appendage length in colder environments (Allen, 1877). Despite receiving far less attention than Bergmann's rule, the same confusion was generated when testing the validity of Allen's rule in ectotherms (Alho et al., 2011; Jin & Liao, 2015; Jin et al., 2006).

Although endotherms maintain their body temperature mainly through metabolism and cease growth after maturation, ectotherms are highly dependent on external sources of heat to achieve their preferred temperature and continue to grow after sexual maturation. Therefore, there may be entirely different mechanisms for ectotherm species, who can be thermoconformers, thermoregulators (Penniket & Cree, 2015), or use other complex processes, unrelated to thermoregulation, that may also cause the variation in ectotherm body size, such as plasticity of growth rates (Walters & Hassall, 2006) and age structure (Liao & Lu, 2012). In addition, lizards generally possess sexual size dimorphism, which also affects body size (Blanckenhorn et al., 2006). Therefore, it would be helpful to consider age and sex in the research of biogeographic rules in ectotherms.

The small, diurnal, toad‐headed lizard *Phrynocephalus przewalskii* (Agamidae) is widely distributed in northern China and adjacent Mongolia (Urquhart et al., 2009) and mainly lives in sand dunes and semi‐desert habitats (Zhao & Liu, 2012). Most females produce one clutch per year, from May to July (Zhao et al., 2011), and the trade‐off between offspring number and size of *P. przewalskii* vary significantly among geographic populations (Wang et al., 2011; Zeng et al., 2013). Males and females usually achieve sexual maturity at a body length of approximately 43 and 45 mm, respectively, in September to October after hatching (Zhao & Liu, 2014). However, the patterns of variation in body size along geographic gradients have not been investigated.

In this study, we explored the variation in the body size of *P. przewalskii*, and the correlation between body size and environmental factors, across a broad geographic area. We aimed to test the validity of Bergmann's rule and Allen's rule in *P. przewalskii* and examine whether age affected the variation in body size. We specifically focused on testing whether: (a) age structure better explains variations in body size than mean ambient temperature; and (b) relative appendage size was affected by mean ambient temperature but not age structure.

## 2 MATERIALS AND METHODS

### 2.1 Ethics statement

Animals were treated in accordance with the guidelines of Ethics Committee of the School of Life Sciences, Lanzhou University, that specifically approved this study.

### 2.2 Sampling sites and sampling method

**FIGURE 1:**
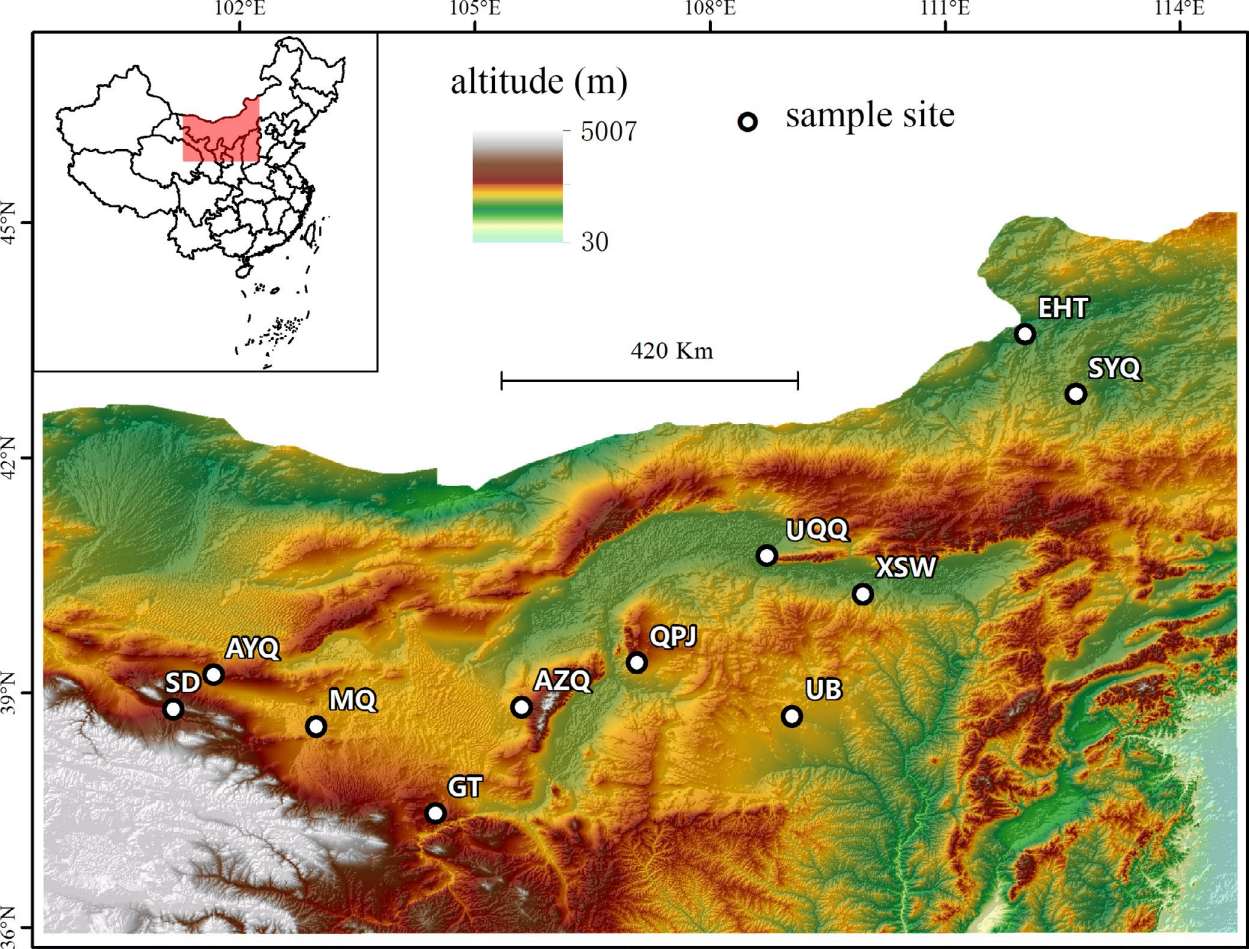
Sampling sites of*Phrynocephalus przewalskii*.*SD* = Shandan, SYQ = Sonid Youqi, UQQ = Urad Qianqi, EHT = Erenhot, UB = Uxin Banner, XSW = Xiangshawan, GT = Gantang, AYQ = Alxa Youqi, MQ = Minqin, AZQ = Alxa Zuoqi, QPJ = Qipanjing. The elevation gradient is represented by graduated color, from green to brown (low to high). The map was downloaded from Earth Resources Observatory and Science (EROS) Center (http://eros.usgs.gov/ #)

A total of 463 lizards were collected from 11 populations along a latitude gradient in northern and western deserts of China in August 2015 (Figure 1). Lizards were captured by hand and were measured immediately. We measured snout‐vent length (SVL), tail length (TL), head length (HL), head width (HW), body width (BW), forelimb length (FL), and hind limb length (LL) of individuals to 0.01 mm, with calipers (Table S1). The sex of each individual was identified through the presence or absence of a hemipenis. Then, the second phalange of the longest toe of the left hind limb was surgically removed and stored in 10% neutral buffered formalin for further treatment. After the wounds had been sterilized with 75% ethanol and staunched with Yunnan Baiyao (a hemostatic agent), lizards were released to the area from which they had been captured.

The geographical information of sampled sites was recorded using a Garmin Oregon E20 handheld GPS unit (Garmin). Five years of climate data for each sample site were collected from nearby climatic stations with the same altitude by the Chinese National Climatic Data Center (CDC). To survey the climate of activity seasons, the monthly means of temperature (°C), rainfall (mm), relative humidity (%), and hours of sunshine (h) were selected. Then, the means of temperature, and the accumulative rainfall and sunshine of activity seasons, which lasted from August to October and next April to July (Liu & Chen, 1996), were calculated (Table 1). As the effect of weather conditions on plant primary productivity had no time delay (Long et al., 2010), we calculated climate factors for each lizard based on its age. For example, for a lizard of 2 years old in 2015, we calculated the mean climate of 2014 and 2015.

**TABLE 1:** Details of the 11 study sites of *Phrynocephalus przewalskii*

Site	Latitude (N)	Longitude (E)	Altitude (m)	Temperature (^o^C)	Precipitation (mm)	Sunshine duration (h)
SD	38.792	101.154	1,895	15.62 ± 0.11	210.00 ± 17.95	1,760.62 ± 30.15
SYQ	42.813	112.671	1,096	15.64 ± 0.21	198.40 ± 31.27	1,956.16 ± 14.21
UQQ	40.754	108.728	1,081	15.94 ± 0.17	238.64 ± 50.39	2,020.30 ± 29.77
EHT	43.578	112.025	964	16.18 ± 0.16	117.54 ± 16.25	2,056.20 ± 17.33
UB	38.704	109.051	1,384	16.23 ± 0.15	213.26 ± 26.02	1,761.12 ± 27.16
XSW	40.262	109.958	1,111	17.09 ± 0.09	267.66 ± 46.42	1,787.32 ± 31.50
GT	37.461	104.495	1,682	17.18 ± 0.15	195.70 ± 24.71	1,713.22 ± 26.00
AYQ	38.817	105.598	1,434	17.48 ± 0.10	211.36 ± 18.17	1,754.12 ± 52.68
MQ	38.572	102.978	1,382	18.18 ± 0.09	114.38 ± 8.24	2,025.80 ± 23.29
AZQ	39.23	101.661	1,502	18.20 ± 0.15	125.36 ± 13.51	2,054.30 ± 17.00
QPJ	39.384	107.072	1,389	18.74 ± 0.10	157.26 ± 16.38	1,917.68 ± 35.02

Note

The climatic data from April to October (corresponding to the activity season of the lizards) over 5 years were collected from the Chinese Climatic Data Center (CDC) and shown as Mean ± *SE*. Population are arranged in ascending order of annual mean temperature. Abbreviations are the same as in Figure 1.

### 2.3 Age determination and growth rate estimation

Skeletochronology was used to produce histological sections for aging. After clearing surrounding tissues of the phalanx, each digit sample was decalcified in a 5% nitric acid solution for 24 hr and stained for 90 min in Harris's hematoxylin. Then, phalanxes were dehydrated using increasing ethanol concentrated solutions and prepared for embedding in small paraffin blocks. Phalanx diaphysis cross sections (13 µm) were obtained using a rotary microtome, then transparentized by xylene. After sealing using neutral gum, slices from each individual were observed with a light microscope and the transparent sections with smaller medullary cavities and the thickest cortical bone were used to count the lines of arrested growth (LAGs).

Von Bertalanffy model (von Bertalanffy, 1938) with a nonlinear regression procedure was used to estimate the growth rate for each sex:

$${\text{SVL}}_{t}={\text{SVL}}_{m}‐\left({\text{SVL}}_{m}‐{\text{SVL}}_{0}\right)\times e^{‐kt},$$

where SVL*_t_* is SVL at age *t*, SVL_m_ is the asymptotic SVL, SVL_0_ is the hatching SVL, and k is a growth coefficient.

The smallest neonate recorded in the literature was set as the hatchling SVL (25.98 mm for both sexes) (Zhao & Liu, 2014). Annual growth rates (*R*) were calculated after (Iturra‐Cid et al., 2010) as:

$$R=k\times ({\text{SVL}}_{m}‐{\text{SVL}}_{t}).$$

### 2.4 Statistical analyses

All measurement data, except for age which was log_10_(age + 1) transformed, were log_10_‐transformed to meet the assumption of parametric tests. All statistical analyses were performed in SPSS 20.0 software (IBM Corporation) using the Type III sums of squares tests. All probabilities were two‐tailed, and results were statistically significant if *p* < .05. All values are presented as means ± *SE*.

To examine overall lizard sizes (McCoy et al., 2006) and reduce the dimensionality of the data, we performed a principal component analysis (PCA) on log_10_ SVL, log_10_ BW, log_10_ HL, and log_10_ HW. We retained only one axis with an eigenvalue larger than 1 from the PCA, which explained 68.4% of the variation (Table S2). The principle component (PC_BS) had a high positive loading on all characters and can be used to represent overall body size. Another PCA on size‐adjusted limb and tail measurements, which was calculated as the residuals from the regression of SVL, was conducted to represent appendage size. Only one principle component (PC_AS), which explained 70.8% of the variation, was retained and was defined as appendage size (Table S3).

Sex and population differences in mean age were tested using a two‐way analyses of variance (ANOVA), with log_10_(age + 1) as a dependent variable, and population and sex as fixed factors. The differences in body size (PC_BS) and appendage size (PC_AS) among populations and between sexes were tested by two‐way ANCOVA, with population and sex set as factors, and age set as the covariate. The same procedure was conducted on the annual growth rate.

As the latitude of sampling sites was negatively correlated with altitude (Pearson: *r* = −.787, *p* < .001), we tested correlations with temperature instead of latitude on PC_BS, for males and females, respectively, to investigate the adherence of *P. przewalskii* to Bergmann's rule. Similarly, a correlation of appendage size with temperature was conducted to test Allen's rule.

To assess the degree to which morphological and environmental variables were correlated, we ran GLMMs for PC_BS and PC_AS. Here, individuals were nested within populations in the model, sex and log_10_(age + 1) were included in the model as fixed factors, and temperature, rainfall, sunshine, and the interaction of sex and log_10_(age + 1) were covariates, whereas the interaction of population and sex were random factors. The same model was used to identify the environmental factors that affect annual growth rates.

## 3 RESULTS

### 3.1 Geographical variation in morphology

Body size (PC_BS) scaled tightly with age for both sexes (Adj *r* ^2^ ≥ .529, *p* < .001 in all cases), whereas PC_AS showed no relationship to age (Male Adj *r* ^2^ < .001, *p* = .765; Female Adj *r* ^2^ < .001, *p* = .309). Even when age was controlled, there still was a significant geographic variation in PC_BS (*F*_10,440_ = 18.647, *p* < .001) and PC_AS (*F*_10,438_ = 8.654, *p* < .001). No significant sexual differences or sex × population interactions were found for any of these traits (*p* ≥ .097 in all cases).

**FIGURE 2:**
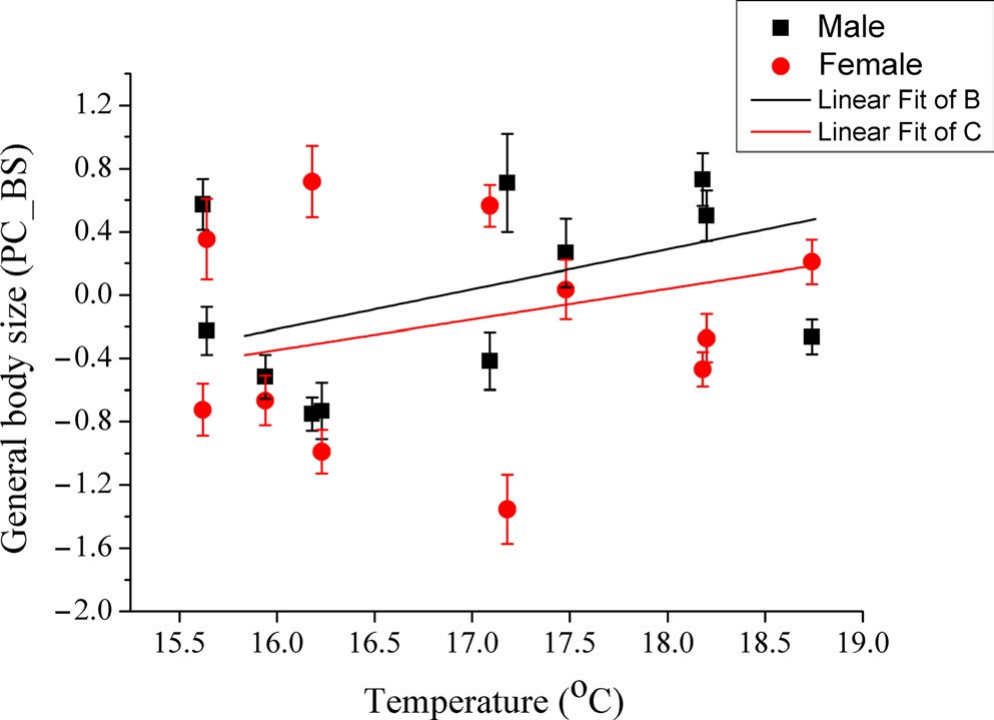
The correlation between general body size of*Phrynocephalus przewalskii*and temperature, for males and females, respectively. A single axis (PC_BS) used from the principal component analysis on log_10_SVL, log_10_BW, log_10_HL, and log_10_HW was general body size. The black squares and black lines represent males, while the red circles and red lines represent females. PC_BS is shown as mean ± *SE*

**FIGURE 3:**
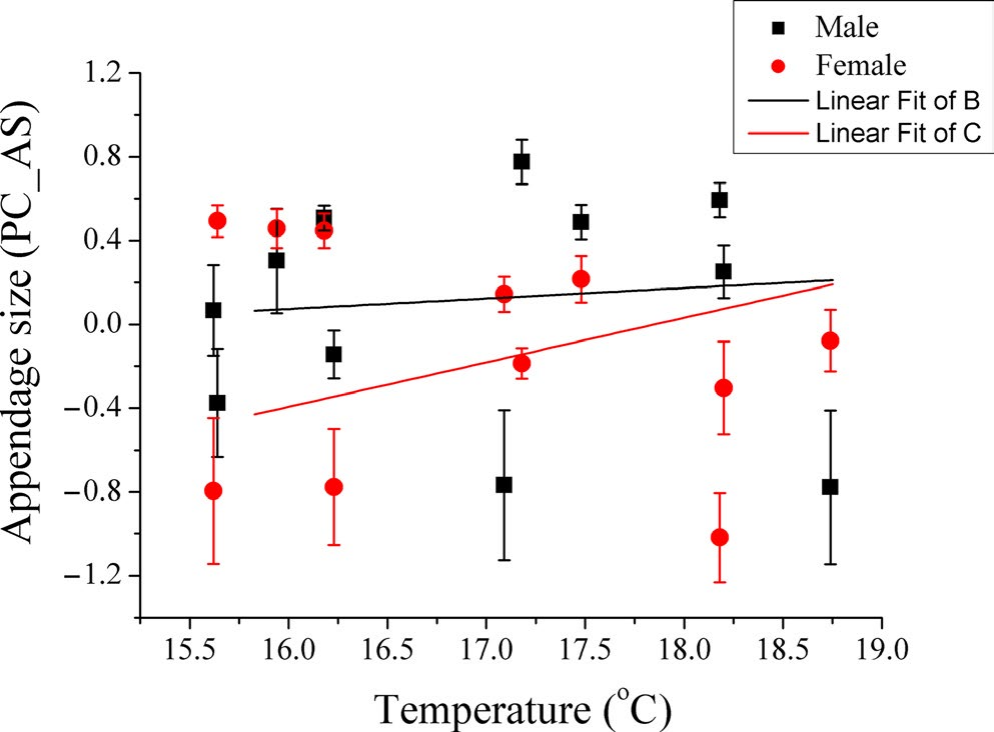
The correlation between appendage size of*Phrynocephalus przewalskii*and temperature, for males and females, respectively. A single axis (PC_AS) used from the principal component analysis on log_10_TL, log_10_FL, and log_10_LL was general body size. The black squares and black lines represent males, while the red circles and red lines represent females. PC_AS is shown as mean ± *SE*

Despite a large proportion of variation being unexplained, correlation analysis indicated that PC_BS significantly increased with increasing temperature for both sexes (male Adj. *r* ^2^ = .051, *p* < .001; female Adj. *r* ^2^ = .032, *p* = .004) (Figure 2). After ages had been added, the fit of the model was significantly improved and the variation trend remained significant for PC_BS (male Adj. *r* ^2^ = .549, *β* = 0.115, *p* = .010; female Adj. *r* ^2^ = .576, *β* = 0.142, *p* = .001). PC_AS showed a significant positive correlation with temperature for females (Adj. *r* ^2^ = .075, *F*_1,227_ = 19.353, *p* < .001) but not for males (Adj. *r* ^2^ = .001, *F*_1,230_ = 0.797, *p* = .373) (Figure 3).

### 3.2 Environmental correlation

The GLMM revealed that PC_BS of *P. przewalskii* was only significantly affected by age (*Z* = 7.008, *p* < .001) but was not significantly affected by sex, temperature, rainfall, or sunshine (*p* ≥ .099 in all cases). PC_AS was affected by age and all the environmental factors previously mentioned, with PC_AS positively correlated with temperature (*Z* = 0.704, *p* = .001) and negatively correlated with age (*Z* = −1.455, *p* = .003), rainfall (*Z* = −0.010, *p* < .001), and sunshine (*Z* = −0.010, *p* < .001). We did not find any significant effect of the interaction between sex and age on any of these measurements (*p* ≥ .923 in all cases).

### 3.3 Geographical variation in age and growth pattern

**FIGURE 4:**
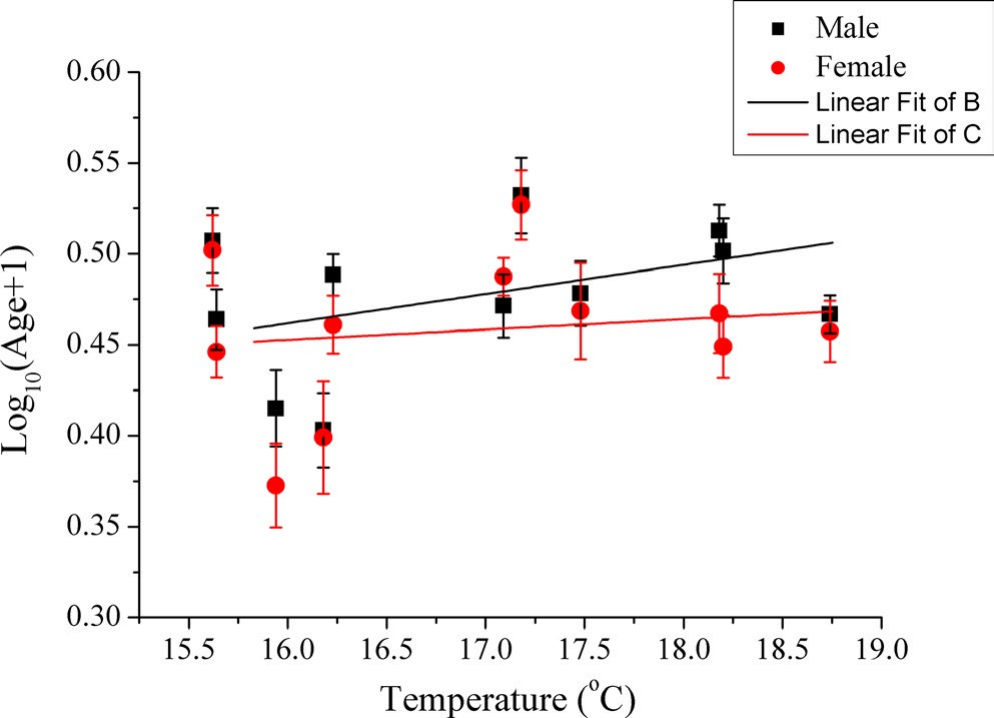
The geographic variation of mean age in*Phrynocephalus przewalskii*. The black squares and black lines represent males, while the red circles and red lines represent females. Mean age is shown as mean ± *SE*

The ages ranged between 1 and 4 years for males and 1 and 3 years for females. There was a significant sexual (*F*_1,441_ = 4.384, *p* = .037) and geographic (*F*_10,441_ = 7.023, *p* < .001) variation in mean age but no population × sex interaction was found (*F*_10,441_ = 0.635, *p* = .784). The GLMM indicated that mean age neither correlated with temperature (*Z* = 0.005, *p* = .542), nor annual rainfall (*Z* = −0.001, *p* = .451), but negatively correlated with sunshine duration (*Z* = −0.001, *p* = .048) (Figure 4).

**FIGURE 5:**
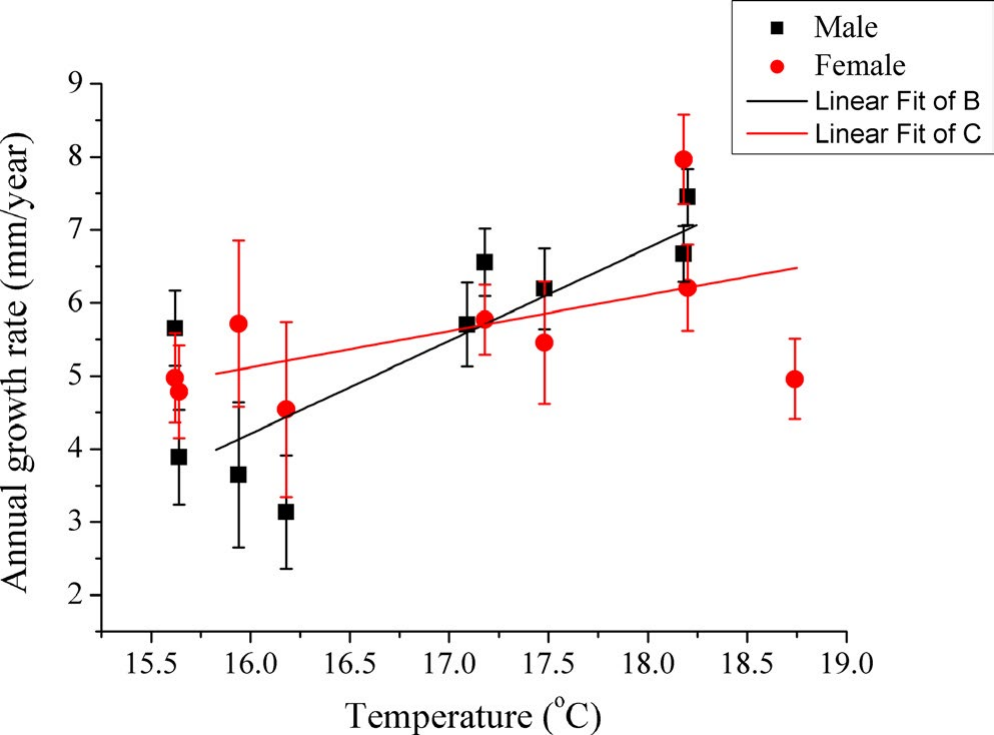
The correlation between annual growth rate of*Phrynocephalus przewalskii*and temperature, for males and females, respectively. The black squares and black lines represent males, while the red circles and red lines represent females. The annual growth rate is shown as mean ± *SE*

As the age classes of some populations was less than three, five models in which *r* ^2^ was less than .5 or the asymmetric SVL was obviously wrong were eliminated in the following analyses (Table 2, Fig S1). After controlling for age, *R* varied significantly between sexes (*F*_1,376_ = 3.858, *p* = .050) and among populations (*F*_8,376_ = 59.975, *p* < .001). We also found a significant interaction between sex and population (*F*_7,376_ = 59.975, *p* < .001). The GLMM indicated that *R* was negatively correlated with age (*Z* = −30.206, *p* < .001), but positively correlated with temperature (*Z* = 0.407, *p* = .014), and the interaction between sex and age was significant (*Z* = 5.742, *p* = .002). As the temperature increased, the *R* of both males (Adj *r* ^2^ = .144, *F*_1,185_ = 32.330, *p* < .001) and females (Adj *r* ^2^ = .023, *F*_1,205_ = 5.770, *p* = .017) increased significantly (Figure 5).

**TABLE 2:** Growth parameters of von Bertalanffy model for *Phrynocephalus przewalskii*

Site	Sex	*R* ^2^	Asymptotic SVL (mm)	*k*
SD	Male	0.691	61.39 ± 3.07	0.636 ± 0.124
	Female	0.822	58.35 ± 1.56	0.771 ± 0.099
SYQ	Male	0.517	52.77 ± 1.27	1.144 ± 0.192
	Female	0.529	53.47 ± 1.80	1.039 ± 0.202
UQQ	Male	0.544	49.85 ± 0.92	1.636 ± 0.267
	Female	0.759	51.86 ± 1.04	1.292 ± 0.138
EHT	Male	0.642	48.47 ± 0.46	1.952 ± 0.196
	Female	0.771	49.00 ± 1.12	1.432 ± 0.235
UB	Male	0.397^a^	64.75 ± 13.87	0.442 ± 0.262
	Female	0.306^a^	52.14 ± 3.34	1.242 ± 0.634
XSW	Male	0.417^a^	57.49 ± 1.49	0.694 ± 0.236
	Female	0.780	131.31 ± 91.75^a^	0.116 ± 0.114
GT	Male	0.816	68.48 ± 5.28	0.406 ± 0.090
	Female	0.721	63.24 ± 4.32	0.513 ± 0.120
AYQ	Male	0.655	60.20 ± 4.06	0.636 ± 0.158
	Female	0.723	55.96 ± 1.70	0.862 ± 0.132
MQ	Male	0.803	66.66 ± 2.70	0.512 ± 0.067
	Female	0.8	67.08 ± 4.12	0.511 ± 0.095
AZQ	Male	0.755	70.25 ± 4.41	0.407 ± 0.070
	Female	0.817	57.46 ± 1.22	0.850 ± 0.083
QPJ	Male	0.096^a^	49.07 ± 1.49	1.788 ± 0.916
	Female	0.711	53.64 ± 1.69	0.886 ± 0.142

^a^ The models which *R* ^2^ < 0.5 or asymmetric SVL was obvious wrong were eliminated in the following analysis. Population are arranged in ascending order of annual mean temperature. Abbreviations are the same as in Figure 1.

## 4 DISCUSSION

Similar to previous studies, which suggested that age determines the altitudinal variation of body size in anurans (Liao & Lu, 2011, 2012), GLMM indicated that only age affected the variation of PC_BS in *P. przewalskii*. The coefficient of determination between temperature and body size was small, which suggested that *P. przewalskii* may not follow Bergmann's rule. Previous studies have also suggested that Bergmann's rule does not seem to be applicable to lizards, as most evidence suggests that lizards show the converse of Bergmann's trend (Ashton & Feldman, 2003), or no trend at all (Pincheira‐Donoso & Meiri, 2013).

The temperature‐size rule is used to explain Bergmann's cline hypotheses that ectotherms grow slower in colder environments and mature later (Angilletta et al., 2004). Our results showed that the growth rates of *P. przewalskii* correlated positively with temperature. However, the decreased annual growth rates in cold areas did not lead to delaying maturation. Therefore, there is no Bergmann's cline in *P. przewalskii*. Food quality can also cause variation in growth rates of ectotherms and effect the body size (Michael et al., 2014). Ectotherms generally mature later at smaller sizes under nutrition limitation but at larger sizes under thermal restriction (Berrigan & Charnov, 1994). Perhaps energy restriction is also involved in the size variation of *P. przewalskii*.

The different selective pressures faced by each sex affect the growth rate after maturation in ectotherms. Females selected by fecundity allocate more energy to reproduction and grow more slowly than males after maturation (Cox & Calsbeek, 2010). Our result also suggested that the growth rate of females decreased faster with increasing age than that of males in *P. przewalskii*. However, previous studies have failed to find a significant difference in the growth rate between the two sexes of *P. przewalskii* after maturation (Zhao & Liu, 2014). Our results indicated that the interaction between sex and population does not affect the body size of *P. przewalskii*. Therefore, further study is needed to uncover the role of sex in body size variation in *P. przewalskii*.

Our result showed that the appendage size of *P. przewalskii* varied between the two sexes, with females following Allen's rule. However, *Phrynocephalus vlangalii* and *Phrynocephalus theobaldi*, which inhabit the Tibetan Plateau, contravene Allen's rule, possibly due to the heat advantage gained during basking (Jin & Liao, 2015; Jin et al., 2006). In contrast to the Tibetan Plateau, thermal restriction in low altitude deserts might be caused by overheating rather than cold. The temperature of the sand surface is much higher than that of the air in low altitude desert (Yan et al., 1997); thus, during our field study, *P. przewalskii* were raised their bodies as high as they could. Hence, for females, a relatively longer appendage in a hot desert may provide an advantage for the maintenance of the preferred body temperature, not only through enhanced heat dissipation but also by reducing the heat gained from the land surface. However, males of *P. przewalskii* compete for mates and a relatively large appendage is advantageous in male–male combat and mate chasing. Therefore, males still maintain a large appendage, even in cold habitats. The GLMM analysis also showed that appendage size was significantly negatively correlated with rainfall and sunshine. In the northern deserts of China, rainfall and sunshine positively affect the primary productivity and height of annual plants (Yan et al., 2013). A relatively short appendage may be advantageous for crawling through closed vegetation (unpublished data).

In conclusion, our results showed that the body size variation in *P. przewalskii* is mainly caused by age structure and the plasticity of growth rate and may show no trend with temperature at all. Females of *P. przewalskii* follow Allen's rule, whereas males neither follow nor contravene Allen's rule. Further studies about the effect of energy restriction, thermal advantage, and sexual selection may be helpful to understand the body size variation in *P. przewalskii*.

## CONFLICT OF INTEREST

We declare that we have no conflict of interest.

## AUTHOR CONTRIBUTIONS

**Wei Zhao:** Resources (equal); software (equal); writing – original draft (equal); writing – review and editing (equal). **Yangyang Zhao:** Validation (equal). **Rui Guo:** Validation (equal). **Yue Qi:** Validation (equal). **Xiaoning Wang:** Validation (equal). **Na Li:** Validation (equal).

## Supporting information

Figure S1

Supplementary Material

## Data Availability

The data underlying this article are available by agreement with our partners.
