# Supplementary figures and images for: Age and annual growth rate cause spatial variation in body size in Phrynocephalus przewalskii (Agamid)
Source: Ecol Evol. 2020 Nov 13;10(24):14189–95. doi: 10.1002/ece3.7013 (PMC7771167; doi:10.1002/ece3.7013)

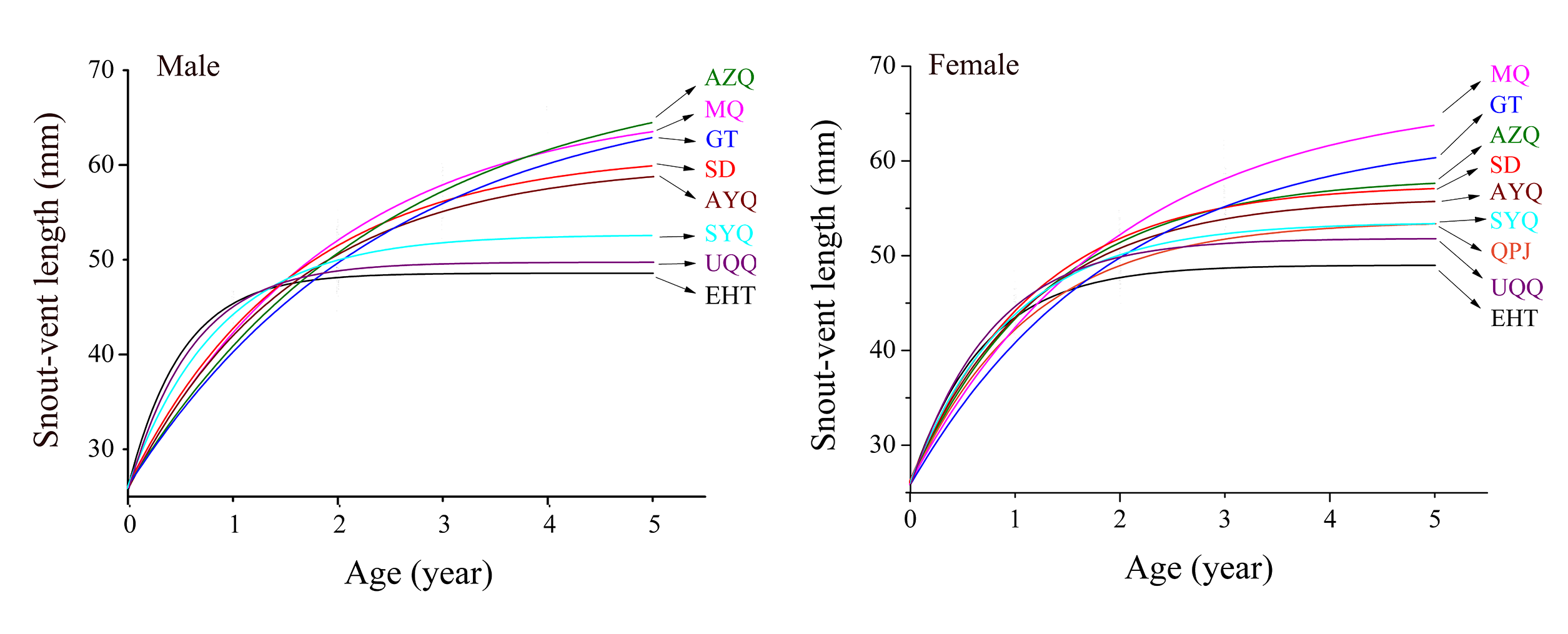

Supplement: Supplementary file 1 — Figure S1 [file ECE3-10-14189-s001.tif]
