# Supplementary material for: Age and annual growth rate cause spatial variation in body size in Phrynocephalus przewalskii (Agamid)
Source: Ecol Evol. 2020 Nov 13;10(24):14189–95. doi: 10.1002/ece3.7013 (PMC7771167; doi:10.1002/ece3.7013)

**Age and annual growth rate cause spatial variation in body size in *Phrynocephalus przewalskii* (Agamid)**

Wei Zhao^1^*, Yangyang Zhao^1^, Rui Guo^1,2^, Yue Qi^1^, Xiaoning Wang^1^, Na Li^1^

**Supplementary information**

Table S1. Morphological measurements and sample size of *Phrynocephalus przewalskii* collected in 2015. All measurements are reported as Mean ± SE, SVL = snout-vent length, from snout to vent; TL = tail length, form vent to the end of tail; BW = body width, the widest point of body; HL = head length, from vent to the anterior edge of the tympanum; HW = head width, the widest point of head; FL = forelimbs length, from the insertion point of the limb to the tip of the foot; LL = hindlimbs length, from the insertion point of the limb to the top of the foot. EHT = Erenhot, GT = Gantang, MQ = Minqin, QPJ = Qipanjing, SD = Shandan, SYQ = Sonid Youqi, UQQ = Urad Qianqi, UB = Uxin Banner, XSW = Xiangshawan, AZQ = Alxa Zuoqi, AYQ = Alxa Youqi.

| Sites | Sex | N | SVL | TL | BW | HL | HW | FL | LL |
| --- | --- | --- | --- | --- | --- | --- | --- | --- | --- |
| EHT | Male | 19 | 46.86 ± 0.4 | 60.93 ± 0.72 | 13.00 ± 0.22 | 12.64 ± 0.34 | 10.54 ± 0.11 | 22.37 ± 0.32 | 36.76 ± 0.58 |
|  | Female | 8 | 46.39 ± 0.7 | 55.19 ± 0.66 | 12.37 ± 0.48 | 11.53 ± 0.3 | 10.00 ± 0.16 | 20.54 ± 0.65 | 33.05 ± 0.81 |
| GT | Male | 18 | 52.95 ± 1.01 | 72.48 ± 1.97 | 14.21 ± 0.4 | 15.65 ± 0.61 | 12.85 ± 0.37 | 25.82 ± 0.51 | 39.94 ± 0.65 |
|  | Female | 19 | 51.99 ± 0.94 | 67.29 ± 0.99 | 15.42 ± 0.63 | 14.89 ± 0.37 | 12.26 ± 0.27 | 24.74 ± 0.34 | 37.92 ± 0.53 |
| MQ | Male | 35 | 53.63 ± 0.75 | 71.92 ± 1.50 | 14.78 ± 0.37 | 13.92 ± 0.33 | 12.85 ± 0.23 | 25.48 ± 0.38 | 39.08 ± 0.60 |
|  | Female | 25 | 53.01 ± 1.11 | 69.47 ± 1.66 | 14.85 ± 0.48 | 13.73 ± 0.48 | 12.89 ± 0.26 | 24.73 ± 0.51 | 38.71 ± 0.91 |
| QPJ | Male | 17 | 48.24 ± 0.61 | 60.91 ± 1.03 | 14.18 ± 0.36 | 12.67 ± 0.18 | 11.35 ± 0.19 | 19.77 ± 1.30 | 28.82 ± 1.86 |
|  | Female | 21 | 48.55 ± 0.57 | 58.01 ± 0.87 | 15.15 ± 0.42 | 12.80 ± 0.23 | 11.08 ± 0.19 | 21.43 ± 0.91 | 33.18 ± 1.24 |
| SD | Male | 19 | 52.50 ± 0.81 | 69.06 ± 1.41 | 14.14 ± 0.33 | 14.55 ± 0.51 | 12.37 ± 0.27 | 22.97 ± 0.82 | 35.79 ± 1.31 |
|  | Female | 20 | 52.35 ± 0.69 | 64.01 ± 1.00 | 16.30 ± 0.53 | 14.36 ± 0.35 | 11.61 ± 0.18 | 24.34 ± 0.41 | 37.48 ± 0.52 |
| SYQ | Male | 25 | 49.38 ± 0.57 | 60.53 ± 0.97 | 14.20 ± 0.28 | 13.58 ± 0.45 | 10.66 ± 0.15 | 20.60 ± 0.82 | 34.19 ± 1.43 |
|  | Female | 27 | 49.55 ± 0.53 | 56.64 ± 0.50 | 13.32 ± 0.23 | 13.13 ± 0.34 | 10.72 ± 0.10 | 18.83 ± 0.62 | 29.55 ± 1.16 |
| UQQ | Male | 16 | 47.98 ± 0.52 | 66.90 ± 1.63 | 13.59 ± 0.32 | 12.50 ± 0.17 | 11.19 ± 0.15 | 21.08 ± 0.76 | 35.14 ± 1.14 |
|  | Female | 18 | 46.97 ± 0.67 | 57.63 ± 0.86 | 13.00 ± 0.36 | 11.49 ± 0.14 | 10.58 ± 0.19 | 18.72 ± 0.82 | 30.67 ± 1.40 |
| UB | Male | 11 | 49.26 ± 0.83 | 59.76 ± 1.05 | 12.02 ± 0.27 | 12.25 ± 0.28 | 10.76 ± 0.36 | 21.66 ± 0.50 | 34.49 ± 0.52 |
|  | Female | 11 | 49.47 ± 0.88 | 64.41 ± 1.77 | 12.28 ± 0.22 | 12.33 ± 0.22 | 10.70 ± 0.18 | 24.05 ± 0.39 | 36.32 ± 0.63 |
| XSW | Male | 18 | 49.27 ± 0.83 | 61.69 ± 1.34 | 12.96 ± 0.29 | 13.04 ± 0.39 | 10.92 ± 0.22 | 18.76 ± 0.98 | 31.16 ± 1.72 |
|  | Female | 12 | 48.50 ± 0.86 | 58.81 ± 1.29 | 12.65 ± 0.45 | 12.02 ± 0.17 | 10.78 ± 0.14 | 18.56 ± 1.09 | 31.46 ± 1.85 |
| AYQ | Male | 18 | 50.92 ± 0.78 | 65.86 ± 1.27 | 14.51 ± 0.49 | 14.04 ± 0.32 | 12.06 ± 0.24 | 25.30 ± 0.33 | 37.07 ± 0.61 |
|  | Female | 21 | 49.63 ± 0.97 | 60.39 ± 0.87 | 15.10 ± 0.48 | 13.25 ± 0.27 | 11.35 ± 0.18 | 23.75 ± 0.42 | 36.46 ± 0.53 |
| AZQ | Male | 33 | 52.24 ± 0.92 | 71.60 ± 1.89 | 14.90 ± 0.3 | 14.14 ± 0.26 | 12.18 ± 0.21 | 22.96 ± 0.67 | 36.33 ± 1.02 |
|  | Female | 36 | 50.16 ± 0.69 | 64.35 ± 2.18 | 15.14 ± 0.32 | 13.42 ± 0.22 | 11.77 ± 0.19 | 21.35 ± 0.60 | 33.76 ± 0.92 |
| Total | Male | 229 | 50.70 ± 0.28 | 66.47 ± 0.57 | 14.06 ± 0.12 | 13.67 ± 0.13 | 11.74 ± 0.09 | 22.69 ± 0.26 | 35.71 ± 0.39 |
|  | Female | 218 | 50.04 ± 0.28 | 62.02 ± 0.55 | 14.45 ± 0.15 | 13.20 ± 0.12 | 11.42 ± 0.08 | 21.97 ± 0.25 | 34.44 ± 0.38 |

Table S2. Summary of the principal component analysis on body size.

| Variable | PC1 | PC2 | PC3 | PC4 |
| --- | --- | --- | --- | --- |
| eigenvalue | 2.739 | 0.591 | 0.407 | 0.263 |
| SVL | 0.867 |  |  |  |
| BW | 0.723 |  |  |  |
| HL | 0.833 |  |  |  |
| HW | 0.878 |  |  |  |
| Variance explained (%) | 68.463 | 14.780 | 10.180 | 6.577 |
| Cumulative variation (%) |  | 83.243 | 93.423 | 100.000 |

Table S3. Summary of the principal component analysis on appendage size.

| Variable | PC1 | PC2 | PC3 |
| --- | --- | --- | --- |
| eigenvalue | 2.126 | 0.770 | 0.104 |
| TL | 0.601 |  |  |
| AL | 0.933 |  |  |
| LL | 0.945 |  |  |
| Variance explained (%) | 70.868 | 25.674 | 3.458 |
| Cumulative variation (%) |  | 96.542 | 100.000 |

Figure S1. The fitted growth curves of *Phrynocephalus przewalskii*. The model was fitted according to von Bertalanffy (1938) for each sex of each population separately. Five models in which r^2^ was less than 0.5 or the asymmetric SVL was obvious wrong were not shown. Abbreviations are the same as in Supplement table S1.


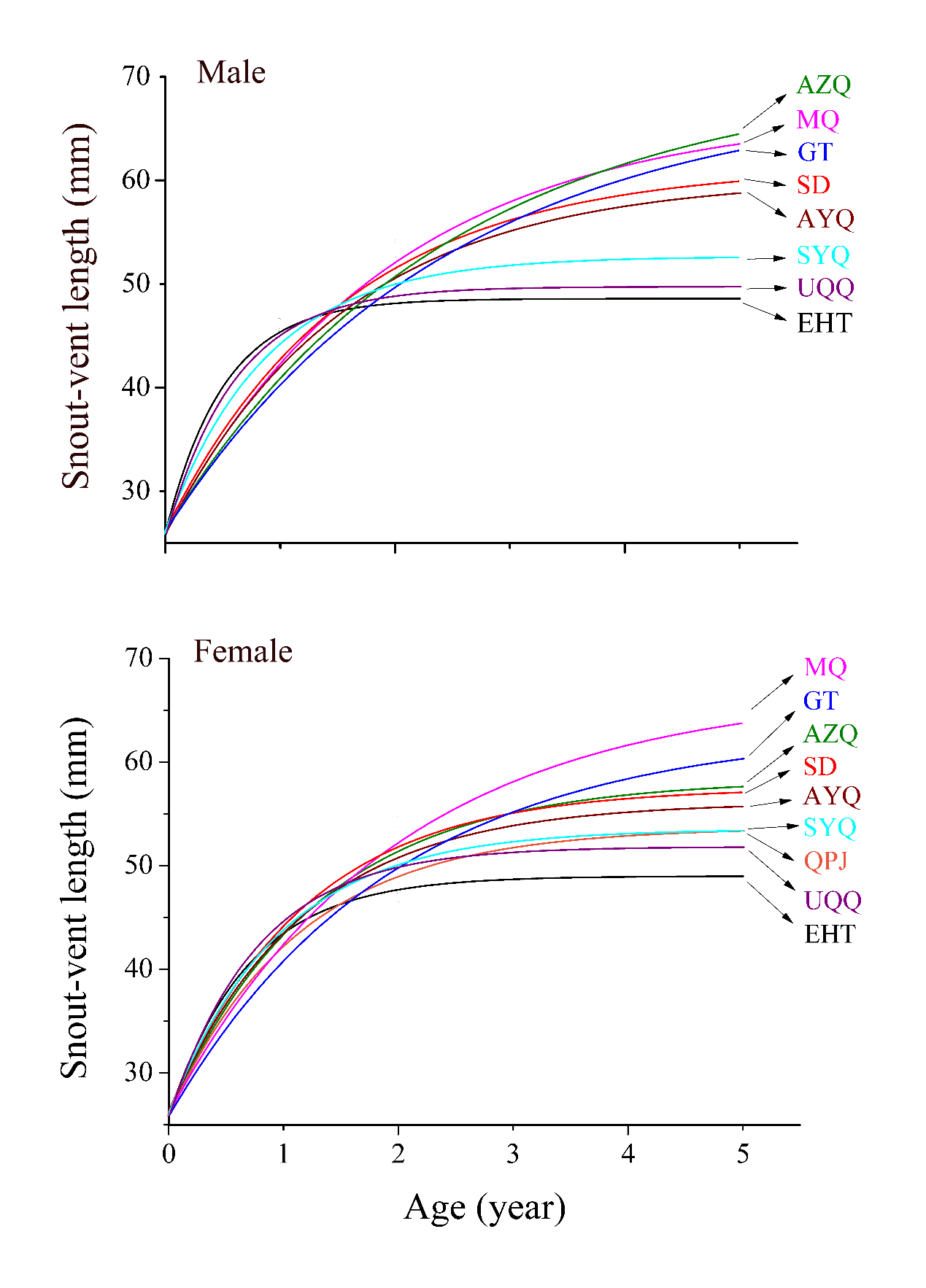

Supplement: Supplementary file 2 — Supplementary Material [file ECE3-10-14189-s002.docx]
